# Understanding machine learning weather prediction by designing a cost-efficient model with knowledge-oriented modules

**DOI:** 10.1038/s41598-025-32366-3

**Published:** 2025-12-15

**Authors:** Minjong Cheon, Jeong-Hwan Kim, Yumi Choi, Yo-Hwan Choi, Seon-Yu Kang, Jeong-Gil Lee, Yoo-Geun Ham, Jin Young Kim, Daehyun Kang

**Affiliations:** 1 Climate and Environmental Research Institute, Korea Institute of Science and Technology, Seoul, South Korea; 2 National Electric Power Control Center, Korea Power Exchange, Naju, South Korea; 3 Department of Environmental Managements, Graduate School of Environmental Studies, Seoul National University, Seoul, South Korea; 4 Applied Science Research Institute, Korea Advanced Institute of Science and Technology, Daejeon, South Korea; 5 Environmental Planning Institute, Seoul National University, Seoul, South Korea

**Keywords:** Engineering, Mathematics and computing

## Abstract

Deep learning-based models are gaining prevalence in global weather forecasting, surpassing the performance of existing numerical models. However, training these models with high-resolution global weather data requires massive computational resources, making it difficult to conduct extensive experiments to understand the model processes. In addition, the reason for region- or variable-dependent accuracy in the machine learning models, along with the extra predictability provided by each component, remains unknown. Therefore, we propose a novel data-driven model named KARINA, which combines Geocyclic Padding and SENet modules with the ConvNeXt backbone to enhance weather forecasting while minimizing training resources. Despite its much lower training cost, KARINA achieved competitive performance compared to the recently developed data-driven models such as Pangu-Weather and GraphCast, while surpassing the numerical weather prediction of ECMWF IFS at a lead time of up to 10 days. The efficient training process and KARINA’s modular structure allow us to demonstrate the effectiveness of Geocyclic Padding and SENet through comprehensive trials. Geocyclic Padding significantly improves the modeling of horizontal advection, while SENet particularly captures the dynamics of atmospheric convection. These findings suggest that incorporating knowledge-oriented techniques can lead to reliable performance. This paper presents a framework for gaining a deeper understanding of the model mechanism and proposes ways to improve machine learning weather prediction models.

**Supplementary Information:**

The online version contains supplementary material available at 10.1038/s41598-025-32366-3.

## Introduction

Numerical Weather Prediction (NWP) has been a cornerstone of weather forecasting over the past decades due to substantial improvements in physics parameterization methods and the incorporation of high-quality observations and data assimilation^1^. However, these prediction systems require a lot of computing resources, to the point that they require supercomputing facilities^2^. These supercomputing facilities consume massive amounts of energy, which is not unrelated to carbon dioxide emissions, a growing concern in the era of climate change^3^. As the global community strives for carbon neutrality, developing more energy-efficient forecasting systems is not just a scientific challenge but an environmental imperative.

In recent years, data-driven weather prediction (DDWP) models, which trained the global atmospheric reanalysis and climate model outputs, have emerged as a transformative alternative. From FourCastNet, the first model to generate forecasts at a 0.25° resolution, to Pangu-Weather, GraphCast, Fuxi, and Aurora, advancements in DDWP were remarkable. Pangu-Weather and GraphCast outperformed the ECMWF high-resolution (HRES) numerical forecasts at lead times within a week. Most recently, Aurora surpassed both cutting-edge DDWP and numerical models, marking a significant step towards a comprehensive Earth system foundation model at a higher resolution of 0.1 degree^4–7^.

Despite of the pioneering development of DDWP models with the Convolutional Neural Networks (CNNs)^8,9^, the development of state-of-the-art global weather models has often trended away from traditional CNNs as a primary backbone, due to several perceived limitations. First, traditional CNNs’ local receptive fields were considered inappropriate for capturing global dependencies^10^. Second, the use of multiple kernels in CNNs was thought to concentrate data relevance around the center of feature maps, making it unsuitable for global weather forecasting^11^. Recently, the ConvNeXt architecture has modernized traditional CNNs by incorporating advanced design principles such as LayerNorm, Inverted Bottleneck, and hierarchical feature extraction stages. ConvNeXt resolves these limitations and has outperformed ViT and Swin Transformer in various vision tasks^12^. It demonstrates high adaptability due to a strong inductive bias that preserves locality, translation invariance, and hierarchical feature learning. Furthermore, by employing larger kernel sizes, ConvNeXt overcomes the disadvantage of local receptive fields to grasp broader spatial characteristics, balancing local and global feature extraction—a significant advantage for global weather forecasting^12^. Despite this enormous potential, the application of modernized CNNs in weather forecasting remains largely unexplored.

This gap highlights a critical challenge in the field. While SOTA models like Pangu-Weather and GraphCast are highly accurate, their development requires prohibitive computational resources for training (e.g., 192 Nvidia V100 GPUs for 64 days for Pangu-Weather). This immense training cost creates a significant barrier to research, effectively turning these powerful models into “black boxes.” It becomes difficult to conduct the extensive sensitivity tests required to understand the model’s inner workings, such as the specific contribution of each architectural component or the reasons behind region- and variable-dependent accuracy. Consequently, a crucial research gap exists: there is a need for a model that is both highly accurate and computationally efficient to train, thereby enabling systematic experimentation to improve our fundamental understanding of MLWP model mechanisms.

This study aims to address this gap by developing and analyzing a novel data-driven global weather forecast model, KARINA (KIST’s Atmospheric Rhythm with Integrated Neural Algorithms). KARINA is designed to be computationally efficient without sacrificing predictive performance, allowing for a deeper investigation into its underlying mechanisms.

The key contributions of this study are multi-faceted. First, we propose a novel and cost-efficient architecture, KARINA, which integrates a modernized Convolutional Neural Network (CNN) backbone (ConvNeXt) with knowledge-oriented, nearly parameter-free modules—Geocyclic Padding and Squeeze-and-Excitation Networks (SENet). Second, we demonstrate that KARINA achieves forecast skill competitive with SOTA models like Pangu-Weather and GraphCast, and surpasses the ECMWF IFS model for lead times up to 10 days, all while requiring only a fraction of the training resources (12 hours on 4 NVIDIA A100 GPUs). Third, through its modular design and efficient training, we conduct comprehensive ablation studies to disentangle the component-specific effects, revealing that Geocyclic Padding effectively models horizontal advection, while SENet enhances the representation of atmospheric convection dynamics. Finally, this work provides a practical framework for developing and analyzing DDWP models, suggesting that incorporating domain knowledge through specialized modules can lead to more reliable and physically coherent forecasts.

## Literature review

This section reviews the progression of major deep learning models, categorized by their core architectural approaches: Graph Neural Networks (GNNs), Convolutional and Transformer-based models, and probabilistic Diffusion models.

### Graph neural network (GNN) based models

GNN-based models represent the Earth as a graph, enabling the direct learning of spatial relationships across the globe. GraphCast^4^, developed by DeepMind, is a landmark model in this category. It maps atmospheric data at a 0.25° grid onto a multi-mesh graph with about 40,000 nodes, allowing efficient message passing. GraphCast can generate global 10-day forecasts in under a minute and has been shown to outperform ECMWF’s High-Resolution Forecast (HRES) on over 90% of the 1,380 evaluation targets.

Following this success, ECMWF introduced the Artificial Intelligence Forecasting System (AIFS)^13^, which is now run experimentally alongside its operational models. AIFS combines a GNN encoder–decoder with a sliding-window Transformer processor, using initial conditions from ECMWF’s assimilation system. In its 2024 update, AIFS was upgraded to 0.25° resolution and has demonstrated forecast skill comparable to physics-based systems, with the added advantage of faster, data-driven inference.

### Convolutional and transformer-based models

Architectures proven in computer vision and sequence processing, such as Convolutional Neural Networks (CNNs) and Transformers, have been widely adapted for learning spatio-temporal patterns in weather data. Pangu-Weather^5^, developed by Huawei, was the first deep learning model to comprehensively outperform the ECMWF operational forecast. It is based on a 3D CNN architecture that incorporates geophysical priors and uses a hierarchical temporal aggregation strategy to effectively control error accumulation over long forecast lead times. Pangu-Weather achieved a 10% reduction in error compared to the IFS while being 10,000 times faster.

In parallel, research has explored Transformer-based architectures for weather prediction. Stormer is built on a standard Vision Transformer (ViT) rather than a Swin Transformer backbone^14^. The model introduces weather-specific adaptations such as pressure-weighted loss functions and a randomized dynamics forecasting objective, which enable it to achieve skillful multi-day forecasts with relatively modest computational cost.

Pushing the scale boundary, Aurora is Microsoft’s large-scale atmospheric foundation model^15^. Aurora is a 3D Swin Transformer with about 1.3 billion parameters, pre-trained on over a million hours of heterogeneous reanalysis and climate simulation data, not just ERA5. It delivers state-of-the-art 10-day forecasts at 0.1° resolution, and as a foundation model, can be fine-tuned for diverse downstream applications such as air pollution prediction at 0.4° resolution. In terms of efficiency, recent work has investigated the Mixture-of-Experts (MoE) paradigm. The EWMoE model integrates sparse MoE layers within a Transformer for global weather forecasting^16^. By activating only a subset of experts for each input, EWMoE balances high parameter counts with efficient inference.

### Probabilistic forecasting with diffusion models

Because weather is inherently chaotic, probabilistic forecasts are critical for quantifying uncertainty. Recent advances have leveraged diffusion models for this purpose. GenCast, from DeepMind, extends GraphCast with a diffusion framework to produce ensemble forecasts. It generates 15-day forecasts across 84 weather variables, with each ensemble member produced in about a minute on a TPU^17^. GenCast outperforms ECMWF’s 50-member ensemble (ENS) across more than 96% of verification targets, including improved skill in predicting extreme events such as tropical cyclones.

Complementing this work, CoDiCast applies a conditional diffusion model to global forecasting^18^. Operating at a 5.625° resolution, it produces 3-day forecasts at 6-hour intervals and provides full uncertainty quantification. While its resolution and forecast horizon are lower than those of GenCast, CoDiCast demonstrates strong deterministic accuracy and highlights how diffusion models can generate distributions of future states, offering a computationally efficient way to assess forecast uncertainty.

## Results

### Model architecture and training data

We introduce a model named Korea Institute of Science and Technology (KIST)’s Atmospheric Rhythm with Integrated Neural Algorithms (KARINA), a global weather forecasting model developed at a 2.5° resolution. KARINA follows a fully convolutional architecture, which maintains the resolution of input data throughout the network and helps the model preserve spatial information, while existing deep learning models often apply a U-shaped architecture through downsampling and upsampling.

**Fig. 1:**
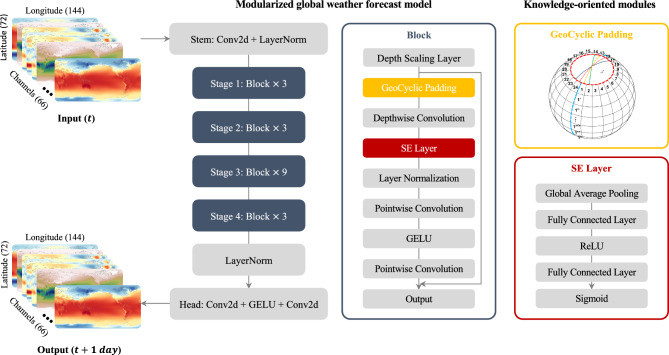
Overall description of KARINA model architecture. It is a modularized data-driven global weather forecast model that merges ConvNeXt with GeoCyclic Padding (denoted as a yellow box; details in Supplementary Figure S1) and SENet (denoted as a red box), targeting daily forecasting with 2.5° horizontal resolution. Features are^3,3,3,9^ stage design, with initial blocks using a kernel size of 7 and depth scaling layers employing a kernel size of 3, channel expansion from 96 to 768, preserving spatial consistency. The maps were generated using the Python Matplotlib (version 3.7.2; http://matplotlib.org/).

Figure 1 displays the architecture of KARINA, which combines ConvNeXt with two modules: GeoCyclic Padding and Squeeze-and-Excitation Networks (SENet). These modules are nearly parameter-free, making our model not only efficient but also enabling it to avoid pooling methods. We introduce ‘Geocyclic Padding’ to improve spatial data handling by reducing distortion from the spherical Earth, which preserves the integrity of atmospheric flows. SENet dynamically recalibrates a large number of channel features^19^, significantly improving atmospheric column processes in vertical pressure levels. With consideration of the physical properties in the dataset, these knowledge-oriented approaches effectively capture complex variable interactions and preserve spatial information with minimal parameter increase.

The European Centre for Medium-Range Weather Forecasts (ECMWF) Reanalysis v5 (ERA5), a reanalysis that covers a global atmosphere at 0.25° horizontal resolution and 37 vertical levels, was used as a dataset^20^. The ERA5 dataset was obtained from the Copernicus Climate Change Service (C3S) Climate Data Store (CDS), interpolated to 2.5° at hourly intervals through the CDS Application Program Interface. Then, the 24-hour data of each day was averaged to daily intervals. We note that the coarser temporal and spatial resolutions of the dataset are beneficial for minimizing high-frequency noise and data uncertainty.

We selected a total of 66 variables consisting of six surface variables and five variables with 12 vertical pressure levels from the surface (1000 hPa) to the lower stratosphere (50 hPa), known as prognostic variables of the Earth’s atmosphere (see the Methods section for the details). The resulting 66 variables were incorporated with a static orography of surface geopotential; then the resulting 67 input variables were organized as a tensor with dimensions (72 × 144 × 67). For the training and evaluation, the 66 variables for 1979-2015 were used as a training set, with the years 2016 and 2017 designated as the validation set and the year 2018 forming the test set.

We produced auto-regressive forecasts at daily intervals with ERA5 for the initial conditions beginning on Monday and Thursday in 2018, the same as the ECMWF Integrated Forecasting System (IFS) for subseasonal prediction. We compare our model against the models available in WeatherBench2 (Pangu-Weather, GraphCast, and ECMWF HRES), validated at 121x240 resolution (1.5°) in 2018^21^. To address a discrepancy from the time interval difference between the WeatherBench2 (6-hourly) and ours (daily) and ensure a fair comparison, we computed daily averages of the skill scores of each model to align with the daily prediction. The average of calculated skill for the successive four 6-hourly steps is considered as one day from the initial condition (e.g., days 1 and 2 of KARINA correspond to averages of 6-, 12-, 18-, 24-hour and 30-, 36-, 42-, 48-hour of the WeatherBench2, respectively). The evaluation metrics employed in the experiments are Root Mean Square Error (RMSE) and Anomaly Correlation Coefficient (ACC).

We conducted an in-depth investigation of KARINA variations, specifically concentrating on assessing the following key meteorological variables: The temperature of the air at 2 m above the surface (T2M), the temperature and, the zonal- and meridional-component of wind at 850 hPa (T850, U850, and V850), the mean sea level pressure (MSLP), the geopotential at 500 hPa (Z500), the specific humidity at 700 hPa (Q700). These variables can explain the comprehensive variability of synoptic-scale weather at the surface and in the low-to-middle troposphere. We first compare the global forecast skill with SOTA models and then compare the regional characteristics and effects of each knowledge-oriented module.

### Performance of KARINA for global weather forecast

**Fig. 2:**
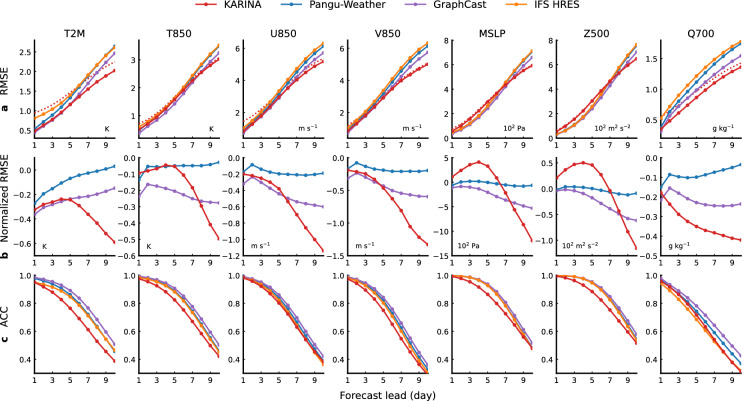
The globally averaged latitude-weighted forecast skills in 2018. Colors indicate KARINA (red), Pangu-Weather (blue), GraphCast (purple), and Integrated Forecasting System (IFS) high-resolution forecast (HRES; orange). (a; first row) RMSE (lower is better), (b; second row), normalized RMSE (i.e., RMSE relative to IFS HRES; negative is gain), and (c; third row) ACC (higher is better). Columns denote each variable: in order from the left column, T2M, T850, U850, V850, T850, MSLP, Z500, and Q700. Specific values for T2M and Z500 is shown in Supplementary Table S1). The dotted red line in the first row shows KARINA downsampled to 1.5°, matching the resolution of the other benchmark models. The figures were generated using the Python Matplotlib (version 3.7.2; http://matplotlib.org/).

Figure 2 assesses the deterministic forecast performance of KARINA compared to the most advanced models in WeatherBench2. The globally-averaged RMSE indicates that the overall weather forecast performance of KARINA proves to be on par with the SOTA machine learning-based and numerical models when evaluated across the seven atmospheric variables (Fig. 2a). Notably, KARINA’s forecasting skill exhibits a slower rate of degradation with increasing forecast lead time compared to the other models. Fig. 2b highlights the model performance as a relative RMSE to ECMWF HRES. While the relative performance of KARINA is similar or less accurate compared to GraphCast until forecast day 5, KARINA performs relatively well on forecast days higher than 5. For a 10-day forecast, KARINA demonstrates a 7.7–17.9% improvement in RMSE over GraphCast. The relative performance is largely dependent on the variables. Among the seven variables validated, T2M, U850, V850, and Q700 are relatively better in KARINA, while T850, MSLP, and Z500 are better in GraphCast until forecast day 7. This variable-dependent performance of KARINA is also consistent in ACC, while GraphCast tends to show the highest ACC values (Fig. 2c).

The competitive performance of KARINA is remarkable, even using much less information with a lower horizontal resolution than the others. This implies that the higher spatial and temporal resolution of data for MLWP, which could impose high-frequency noise and uncertainty of reanalysis data, may be less necessary for the medium- and long-range forecast. On the other hand, the relatively better performance of the cutting-edge deep learning models (Pangu-Weather and GraphCast) until five days reveals the importance of high-resolution datasets for short-term weather forecasts. This indicates that utilizing the efficient model, KARINA, could potentially enhance the forecast performance for predictions extending beyond 10 days such as a subseasonal time scale. We note that KARINA consistently shows reliable skill scores for all 66 variables and stable recursive iterations for more than six months (Supplementary Figs. S2 and S3).

### Effectiveness of model component: Regional characteristics

Here, we further investigate the role of essential components in KARINA, such as Geocyclic Padding and SENet, which significantly improve global weather forecasting accuracy (Supplementary Table S2). To quantify the impact of each module on the model’s performance, we employed a denial study methodology, where the effectiveness of the system was evaluated with and without each respective module. Three versions of the KARINA model were taken into consideration in our analysis: ‘KARINA’ ‘KARINA w/o pad’ and ‘KARINA w/o SENet’, which refer to a model including all components as in Fig. 2, and two models excluding each of GeoCyclic Padding and SENet, respectively. Based on knowledge-based examinations, we elucidate the region-dependent skills and effects of each module, aiming to better understand the essential processes for improving MLWPs.

**Fig. 3:**
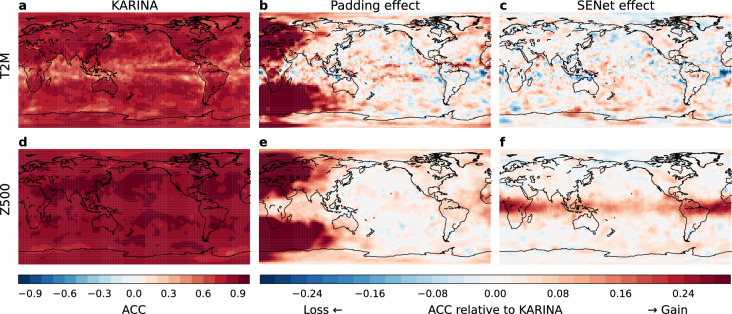
Spatial distribution of ACC on forecast day 5 for T2M and Z500, and the effects of GeoCyclic Padding and SENet. (**a**, **d**) ACC of KARINA for T2M and Z500. (**b**, **e**) ACC difference between KARINA and KARINA w/o pad (i.e., Effect of GeoCyclic Padding) for T2M and Z500. (**c**, **f**) same as b and e, but for KARINA w/o SENet. In the second and third columns, positive (negative) values indicate a gain (loss) in forecasting performance due to the application of each module. The maps were generated using the Python Matplotlib (version 3.7.2; http://matplotlib.org/).

Figure 3 compares spatial distributions of ACC of T2M and Z500 on forecast day 5 in 2018. The results show that KARINA demonstrates outstanding performance in most regions (Figs. 3a and 3 d). ‘Padding effect’ and ‘SENet effect’ denote the skill difference between KARINA and its ablated versions without GeoCyclic Padding and SENet, respectively. The trained model without each module exhibits noticeable regional characteristics. The GeoCyclic Padding significantly improves the accuracy at the image boundary, being effective at both the meridian and the poles (Figs. 3b and 3e). This improvement is particularly evident at the west edge of the image in the mid-latitude, which can be attributed to the horizontal advection by mid-latitude westerly jet that transports the atmospheric memory from west to east. On the other hand, the skill difference is less distinguishable in the east edge, as the advection from the east is generally less important, except in the equatorial region where the easterly atmospheric flow dominates. These improvements propagate inward from the edges as the forecast day increases (Supplementary Fig. S4), indicating that the impact of horizontal advection is effectively considered with the GeoCyclic Padding. The periodic padding at the longitude direction was commonly used in the other MLWP models, while the latitude direction padding at the polar cap is unique in the GeoCyclic Padding. Additionally, a specific experiment was conducted to investigate the latitude direction padding effect on the northern and southern edges, which showed significant improvements in the polar regions (Supplementary Fig. S5). Meanwhile, the impact of the periodic padding at longitude direction is quantitively more significant than that of the padding at latitude direction.

In Figs 3c and 3f, the impact of SENet reveals distinct region- and variable-specific characteristics. The inclusion of SENet results in a marginal improvement for T2M, whereas Z500 shows substantial improvement, particularly in the equatorial region. In contrast to the mid-latitudes, where dynamics are dominated by horizontal advection from background flows, atmospheric convection coupled with vertical motion plays a more significant role in the tropics due to the weak horizontal temperature gradient^22^. This regional disparity in skill improvement suggests that the channel integration with SENet enhances the model’s ability to represent atmospheric column processes more realistically. Likewise, the skill improvements for both specific humidity and geopotential by SENet are most prominent at vertical levels between 700 and 500 hPa (Supplementary Fig. S6). This corresponds to the atmospheric layer where moist convection is a significant driver of perturbations and diabatic heating^23^. This argument is further supported by an analysis of the co-varying vertical profile of the specific humidity anomaly in the convective region, which becomes more realistic with the implementation of SENet (Supplementary Fig. S7).

**Fig. 4:**
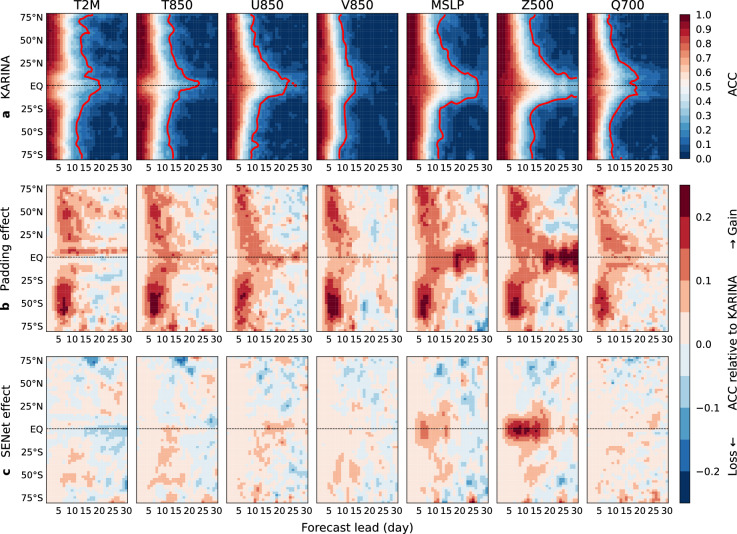
The ACC skill as a function of forecast lead days (x-axis) and latitude (y-axis) for the 7 meteorological variables (T2M, T850, U850, V850, MSLP, Z500, and Q700), and the effects of GeoCyclic Padding. (**a**) Longitudinally averaged ACC of KARINA. The solid red lines indicate two-tailed 95% confidence intervals, derived by Student’s T-test. (**b**) ACC difference between KARINA and KARINA w/o pad (i.e., Effect of GeoCyclic Padding). (**c**) same as b, but for KARINA w/o SENet. In the second and third rows, positive (negative) values indicate a gain (loss) in forecasting performance due to the application of each method. The figures were generated using the Python Matplotlib (version 3.7.2; http://matplotlib.org/).

In Fig. 4, the ACC skill of KARINA and differences with the ablation models are further examined until longer integrations (day 30) as a function of latitude. In the KARINA model, ACC higher than 0.5 persists within a week in the mid- and high-latitudes, while it lasts much longer near the equator. The lower skill in the higher latitudes can be related to the larger non-linear baroclinicity, which is consistently discerned in numerical model forecasts^24^.

In Fig. 4b, the effect of GeoCyclic Padding is prominent in the higher latitude in forecast lead time of less than ten days, as in Fig. 3, while the effect is highlighted near the equator during longer forecast days. This indicates that the improved error from the edge can gradually influence to the image center, resulting in an enhanced forecast skill toward the tropics in longer forecast days. In Fig. 4c, a marked effect of SENet is shown in the tropics, particularly in a few variables such as MSLP, Z500, U850, and Q700. Additionally, relatively minor improvements were discerned in the southern hemisphere in most variables. These findings imply that deep learning models may require specialized modules designed to target the specific dynamical processes predominant in certain regions..

### Effectiveness of model component: Extreme cold and heat events

**Fig. 5:**
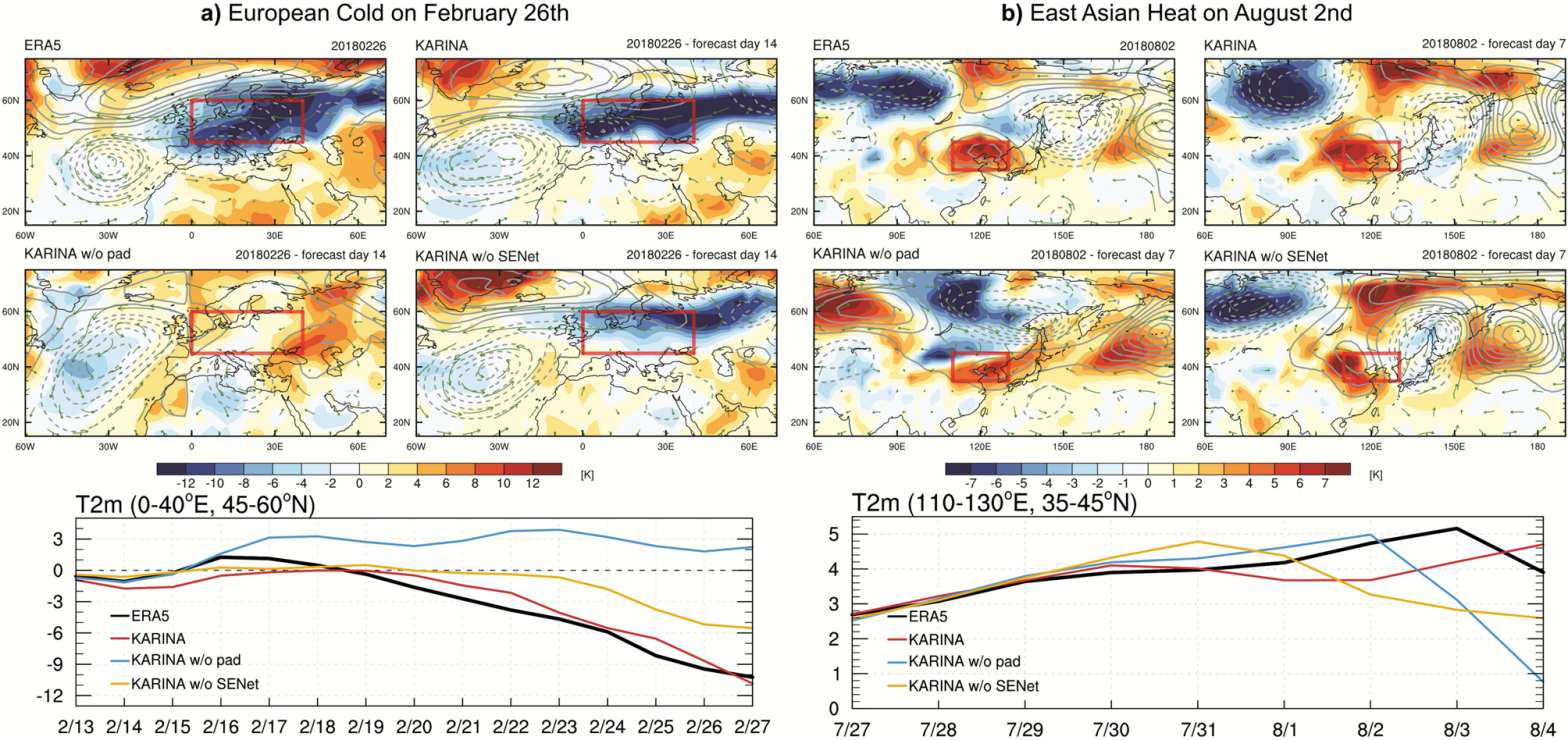
Case study for the European extreme cold in February 2018 and the East Asian heat wave in August 2018. (**a**) Anomalous T2M (shaded), Z500 (contour), wind vectors of U850 and V850 on 26th February 2018 for ERA5 and KARINA forecasts from the initial condition on 12th February. The bottom graph shows area-averaged T2M in western Europe (the red boxes:0-40E; 45-60N). (**b**) Same as (**a**) except for the anomalies on 2nd August 2018 for ERA5 and KARINA forecasts from the initial condition on 26th July. The bottom graph shows area-averaged T2M in East Asia (the red boxes: 110-130E; 35-45N) The maps were generated using the NCAR Command Language (version 6.6.2; 10.5065/D6WD3XH5).

As in the skill score metrics, the effect of each model component is compared to two extreme events in 2018: an extreme cold in Europe and an extreme heat in East Asia. Fig. 5a shows an anomalous state during the extreme cold spell in western Europe on February 26th, 2018. The cold anomalies of less than −10K are discerned in the continental regions with positive (negative) Z500 anomalies over Greenland (Northern Atlantic Ocean), which correspond to the negative phase of the North Atlantic Oscillation (NAO)^25^. The KARINA model reproduces the spatial features of cold temperature anomalies coupled with Z500 anomalies and low-level wind circulation during the negative NAO, even when we present the model output of forecast day 14. The comparable absolute amplitude of temperature anomalies indicates that the model is capable of capturing the strength of large-scale extreme events. On the other hand, KARINA without GeoCyclic Padding cannot reproduce the cold anomalies with noticeable distortion at 0º meridian. This result indicates the necessary role of GeoCyclic Padding in representing the spherical earth authentically. The model without SENet also reproduces realistic features of cold anomalies and negative NAO patterns, while its amplitude is relatively weaker than KARINA. The evolution of area-averaged temperature anomalies in Western Europe consistently shows a better simulation of the cold event in KARINA.

In Fig. 5b, a heat extreme event in East Asia is shown in the same format except for forecast day 7, which is displayed due to the lower model predictability in summer than in winter. The extreme heat anomaly in northeast China and Korea was coupled with high-pressure anomalies on August 2nd, 2018. KARINA model forecast reproduces the major feature of the heat wave and wave train at higher latitude. Although KARINA without GeoCyclic Padding reproduces the anomalous warm pattern near Korea, its spatial distribution does not coincide with the wave train in the higher latitude. Overall, KARINA without SENet reproduces the spatial distribution well enough, but the extreme heat event in the domain is less persistent than that of KARINA.

The examinations of two extreme cases reveal that KARINA is capable of capturing extreme events with effective model components for medium-range forecasts, highlighting the usefulness of the model output. Furthermore, implementing the model components has an essential role in improving extreme events as well as globally-averaged skills.

The longer-range prediction for this extreme cold event were further assessed with the ensemble predictions based on bred vector method. Ensemble prediction provide a reduced error with increasing ensemble up to 51 members (Supplementary Figure S8). The ensemble spreads provide a systematic evolution of the extreme event with that of the North Atlantic atmospheric waves (Supplementary Figure S9). This result implies that KARINA successfully trained the dynamical process for developing cold extremes, which is potentially useful tool for understanding various events with process-oriented diagnostics.

## Discussion

Recent studies have indicated that a skillful deep learning-based weather prediction model provides more accurate forecasts while consuming less time and fewer computational resources compared to typical numerical models for equivalent predictions. However, due to the extensive nonlinear computations performed during training, understanding the interactions within the model is still challenging. Furthermore, those deep learning models still incur high computation costs for training, making it difficult to run multiple experiments to investigate the role of each module.

Therefore, we propose a novel machine learning model for global weather forecasting, named KARINA, which addresses the dual challenges of computational efficiency and forecasting accuracy through extensive experiments. Using a CNN-based approach, we can incorporate parameter-free modules, such as Geocyclic Padding and SENet to capture the physical background in atmospheric variables at a lower computational cost. Those modules are vital to KARINA’s achievement, providing notable enhancements in modeling Earth’s atmosphere, particularly around the image edges and the equatorial regions, respectively. Moreover, CNNs’ high inductive bias enables them to successfully learn spatial traits, which is advantageous with lower resolution data and fewer datasets.

KARINA excelled in global weather forecasting for lead times up to 10 days, showing performance competitive with the high-resolution deep learning-based models known to outperform the ECMWF HRES forecasts. This suggests that KARINA effectively improves reliability in region-specific sensitivity from its components and ensures their physical coherence during forecasts. These results highlight the need to utilize knowledge-oriented methods to increase the dependability of weather forecasting models over long timelines, as they demonstrate that incorporating realistic physics can lead to more accurate forecasts. This study provides a framework that enables a more comprehensive understanding of the model’s inner workings and identifies potential directions for enhancing machine learning weather forecasting models. All current machine learning models for global weather prediction, including our model, still need to be thoroughly explained in terms of why the model was trained to accurately represent physical processes and achieve better skill scores. It would be beneficial to develop a modularized model to address this and to enable various applications.

## Methods

### Data description

The ECMWF Reanalysis v5 (ERA5) was used as a training dataset in this research^20^. Six surface variables (2m temperature, Mean sea level pressure, Surface air pressure, Total column vertically-integrated water vapor, Skin temperature, TOA incident solar radiation) and five variables (Zonal wind, Meridional wind, Temperature, Specific humidity, Geopotential) with 12 vertical pressure levels (1000, 925, 850, 800, 700, 600, 500, 400, 300, 200, 100, 50) were used as input and output variables (Supplementary Table S3). With the 66 variables acquired in hourly intervals, an interpolated dataset to daily average at 2.5° horizontal resolution is organized as a tensor with dimensions (72 × 144 × 66). We noted that the coarser temporal and spatial resolutions help exclude high-frequency noise and reduce uncertainty in data accuracy, thereby enabling training on more salient features crucial for global weather prediction if high-resolution data is not necessary for large-scale features^26^. A previous experiment proved that employing the 66 variables besides orography is far more successful than FourCastNet’s 20 variables^27^. For training and evaluation, the dataset spanning from 1979 to 2015 serves as the training set, with the years 2016 and 2017 designated as the validation set and the year 2018 forming the test set.

### KARINA description

In our study, we refine the architecture of ConvNeXt to develop KARINA, an iteration that enhances ConvNeXt’s performance for weather forecasting at a 2.5° resolution. Our model incorporates two key innovations tailored for the ERA5 dataset: the introduction of Geocyclic Padding to preserve geographical continuity across the 0° and 360° meridians and the poles and the integration of SENet for dynamic feature recalibration. SENet improves the model’s focus on relevant weather variables, while Geocyclic Padding addresses the challenge of representing the Earth’s spherical geometry, ensuring the integrity of horizontal atmospheric fluxes for global weather predictions.

### Baseline: ConvNeXt

ConvNeXt stands as a significant evolution in convolutional neural network algorithms, drawing inspiration from the transformative success of the Swin Transformer architecture^12^. Swin Transformer, known for its efficiency and scalability in processing image data through a hierarchical transformer model, has set new benchmarks in computer vision^28^. ConvNeXt got modernized CNNs by first using sophisticated training techniques from ViTs, such as the AdamW optimizer, longer training schedules, and increased data augmentation. It also adopted the macro architecture by modifying stage compute ratios and replaced the typical ResNet stem with a “patchify” stem, which increased efficiency and scalability.

Inspired by ResNeXt, the ConvNeXt also utilized grouped convolutions and increased network width to balance accuracy and computational cost. An inverted bottleneck design introduced in the MobileNetV2 was also applied, which improved performance even more. The ConvNeXt was also implemented with bigger convolutional kernels, deciding on a 7x7 kernel for best results. On a micro-design level, they replaced ReLU with GELU, decreased the number of activation functions, and replaced BatchNorm with LayerNorm, bringing the network closer to Transformer principles^12^. This fusion allows ConvNeXt to benefit from the adaptability and context-aware processing capabilities of transformers while maintaining the efficiency and locality advantages of CNNs.

Stem Layer: The architecture begins with a Stem layer, a starting point for feature extraction. This layer is a 2D convolutional layer with kernel size 3, stride 1, and padding 1, a configuration that crucially maintains the spatial dimensions of the input. Post the convolutional operation in the stem layer, a layer normalization is applied to stabilize the network by normalizing the features emerging from the convolution, preparing them for subsequent layers^29^.

Depth Scaling Layer: Following the Stem layer, our architecture features a series of depth scaling layers, organized into three stages within a loop that iterates over four distinct ranges. Unlike the conventional downsampling layers used in the original ConvNeXt model, which reduces spatial dimensions, our approach focuses on increasing channel depth while preserving the original spatial dimensions of the input data^12^. LayerNorm is applied at the beginning of each depth-scaling stage to normalize the inputs, followed by convolutional layers that progressively increase the channel dimensions. Specifically, the channel dimensions increase from 96 to 192 in the first stage, from 192 to 384 in the second stage, and from 384 to 768 in the final stage. Each stage starts with LayerNorm, ensuring normalized inputs for the subsequent convolutions that perform the channel enlargement. Each layer in our network is configured with a kernel size of 3, a stride of 1, and padding that preserves the original input size, ensuring that spatial dimensions are consistently maintained.

Block Layer: After the depth scaling layer, our architecture introduces a depthwise convolutional layer with a kernel size of 7 and padding width of 3, utilizing our Geocyclic Padding technique. This layer processes each input channel independently to enhance spatial feature extraction efficiency. Subsequently, a Squeeze-and-Excitation (SE) layer, accompanied by layer normalization, is applied to refine channel-wise features by selectively emphasizing important channels and diminishing less relevant ones. Following layer normalization, the architecture employs two pointwise convolution layers: the first expands the channel dimensions by a factor of four. In contrast, the second restores them to their original size, effectively transforming channel-wise feature representations. The inclusion of the Gaussian Error Linear Unit (GELU) activation function is added^28^, and a learnable scaling parameter is also utilized to adjust the output based on the feature’s significance. The model also integrates residual connections and drop path regularization to facilitate information flow and mitigate overfitting, respectively^30,31^. The ‘depths’ array further details the structure’s complexity, which specifies the number of Block instances in each stage, exemplified by^3,3,3,9^ for a four-stage design.

The final stages include a first convolutional layer for further feature processing, maintaining the channel dimensions, and a second convolutional layer, which acts as the prediction head and maps the features to the same feature shape as the input dataset, effectively preparing the network for prediction.

### SENet

Incorporating the SENet into weather forecasting models effectively enhances their ability to handle and interpret the complexities of multi-channel datasets. The SENet architecture, with its global average pooling followed by a bottleneck of fully connected layers with Rectified Linear Unit (ReLU) and sigmoid activation function, allows the model to focus dynamically on the most relevant features across these channels. Since SENet’s simplified architecture prevents the performance increase from the expense of excessive computing load, it is a useful addition to high-dimensional data processing in meteorological models^32^. By emphasizing significant features and suppressing lesser ones, SENet enhances model predictions of complex weather patterns owing to the nonlinear interactions among variables^33^. Within the KARINA model, pointwise convolution is used for efficient channel integration and dimensionality refinement. The following integration of a SENet effectively modifies the weight assigned to every channel, allowing the model to concentrate more on relevant channels. This method is quite similar to the variable aggregation strategy developed by Nguyen et al., where in cross-attention between channels, the approach minimizes processing demands while fostering a unified, context-aware representation at each spatial point^34^.

### Geocyclic padding

GeoCyclic Padding is a technique tailored for use with Convolutional Neural Networks (CNNs) on geospatial data, like the ERA5 dataset. This padding approach addresses the longitudinal continuity of the Earth by implementing circular padding along the horizontal edges of the input tensor, effectively connecting the 0° meridian with the 360° meridian. This mirroring of the Earth’s geography ensures a coherent representation of longitudinal direction, facilitating a continuous transition at the east-west edge. For the north-south edges, which correspond to the North and South poles, the method involves selecting a row from the circularly padded tensor, bisecting it at 180° longitude, and reordering the segments to reflect the data’s polarity. This step is crucial for accurately simulating the continuity at the poles and minimizing the common distortions found in global map projections. By mirroring the Earth’s spherical geometry, GeoCyclic Padding allows for seamless transitions across both latitudinal and longitudinal boundaries, thus eliminating the artificial discontinuities of the traditional zero padding. This approach minimizes distortions at the poles commonly found in global map projections by accurately maintaining the Earth’s surface continuity in the model through the use of antipodal longitudinal data for padding^35^.

Scher and Messori suggested Polar Padding, which was a pioneering solution specifically designed for handling the latitude-longitude grid of ERA5 by separately processing the Southern and Northern Hemispheres, thereby doubling the model’s trainable parameters^36^. Unlike Polar Padding, GeoCyclic Padding applies a consistent padding technique across the dataset, ensuring spatial continuity without differentiating between hemispheres, presenting a more resource-efficient method. This approach avoids the significant parameter increase from the hemisphere-specific strategy, making GeoCyclic Padding an attractive option for ERA5 datasets.

### Training

We used daily-mean ERA5 data from 1979 to 2015 in the first training phase of our forecasting model, which was intended for one-day-ahead forecasts. Our approach was similar to that of FourCastNet^6^. With the addition of orography data, this extensive dataset (66 variables) allows for an in-depth evaluation of the topographical effects on weather patterns. Employing the KARINA model, our approach analyzed daily datasets formatted as $X(t)$, where $k$ represents the time index with daily intervals. Specifically, the KARINA model was adeptly trained to predict the weather state one day ahead, $X(t+1)$, from the current day’s state, $X(t)$, based on labeled data ${X}_{label}(t+1)$. The training procedure, conducted over 150 epochs with a learning rate of 0.001 and employing the AdamW optimizer in conjunction with a cosine learning rate adjustment, was efficiently completed in less than 12 hours with 4 NVIDIA A100 GPUs (Supplementary Tables S4 and S5).

### Evaluation metrics: RMSE, ACC

In this study, we followed the WeatherBench2 evaluation protocol to fairly evaluate the performance of KARINA against other models^21^. We employed Root Mean Square Error (RMSE), Anomaly Correlation Coefficient (ACC). The global mean of all metrics was calculated using latitude weighting. The latitude weight at latitude ${\varphi }_{i}$, $A({\varphi }_{i}$), is defined as follows:

1 $$A\left({\varphi }_{i}\right)={N}_{lat}\frac{\mathrm{cos}{\varphi }_{i}}{{\sum }_{i=1}^{{N}_{lat}}\mathrm{cos}{\varphi }_{i}}$$

where ${N}_{lat}$ represents the number of grid points in the latitudinal direction.

The ${RMSE}_{l}$ between ground truth (i.e., ERA5) ${y}_{t,i,j}$ and forecast ${\widehat{y}}_{t,l,i,j}$ for lead time $l$ is defined as follows:

2 $${\mathrm{RMSE}}_{l}=\sqrt{\frac{1}{{N}_{time}{N}_{lat}{N}_{lon}}\sum_{t=1}^{{N}_{time}}\sum_{i=1}^{{N}_{lat}}\sum_{j=1}^{{N}_{lon}}A({\varphi }_{i}){({\widehat{y}}_{t,l,i,j}-{y}_{t,i,j})}^{2}}$$

where ${N}_{time}$ and ${N}_{lon}$ denote the number of samples and the number of grid points in the longitude direction, respectively.

The ${ACC}_{l}$ for lead time $l$ is computed as the Pearson correlation coefficient:

3 $${\mathrm{ACC}}_{l}=\frac{1}{{N}_{time}}\sum_{t=1}^{{N}_{time}}\frac{\sum_{i=1}^{{N}_{lat}}\sum_{j=1}^{{N}_{lon}}A({\varphi }_{i})({\widehat{y}}_{t,l,i,j}-\overline{{\widehat{y} }_{t,l,i,j}})({y}_{t,i,j}-\overline{{\mathrm{y} }_{t,i,j}})}{\sqrt{\sum_{i=1}^{{N}_{lat}}\sum_{j=1}^{{N}_{lon}}A\left({\varphi }_{i}\right){\left({\widehat{y}}_{t,l,i,j}-\overline{{\widehat{y} }_{t,l,i,j}}\right)}^{2}\sum_{i=1}^{{N}_{lat}}\sum_{j=1}^{{N}_{lon}}A\left({\varphi }_{i}\right){\left({y}_{t,i,j}-\overline{{\mathrm{y} }_{t,i,j}}\right)}^{2}}}$$

where the upper bar denotes the climatology calculated from the first three harmonics of the climatological seasonal cycle to exclude covariance of seasonality in calendar days.

### WeatherBench2

Motivated by the significant improvements in AI since the release of the first WeatherBench, WeatherBench2 is an updated benchmark for data-driven global weather forecasting. It offers training data, baseline data, ground truth, and an open-source assessment framework. It additionally includes an updated website that features the latest measurements and cutting-edge models. It provides a strong framework for evaluating novel methods against operational baselines and is designed to be compatible with the operational forecast assessments utilized by weather centers^21^. The deterministic skill scores of Pangu-Weather, GraphCast, and ECMWF HRES, validated at 121x240 resolution (1.5°) in 2018, were obtained from the WeatherBench2 website. The skills of each successive four 6-hourly steps from the initial condition was considered as one day prediction.

## Supplementary Information

Supplementary Information.

## Data Availability

The ERA5 dataset used in the experiment is available at [https://cds.climate.copernicus.eu/cdsapp#!/dataset/reanalysis-era5-single-levels](https:/cds.climate.copernicus.eu/cdsapp). The WeatherBench2 skill score dataset is available at baseline datasets through https://github.com/google-research/weatherbench2?tab=readme-ov-file.

## Code availability

The code used for training KARINA is available at https://zenodo.org/records/11439011.
