# Supplementary material for: Understanding machine learning weather prediction by designing a cost-efficient model with knowledge-oriented modules
Source: Sci Rep. 2025 Dec 15;16:2413. doi: 10.1038/s41598-025-32366-3 (PMC12819519; doi:10.1038/s41598-025-32366-3)
Supplement: Supplementary file 1 — Supplementary Information. [file 41598_2025_32366_MOESM1_ESM.docx]

**Supplementary Information**

**On**

**Understanding data-driven weather forecasting by designing a cost-efficient model with knowledge-oriented modules**

Minjong Cheon^1^, Jeong-Hwan Kim^1^, Yumi Choi^1^, Yo-Hwan Choi^1,2^, Seon-Yu Kang^1,3^, Jeong-Gil Lee^1,3^, Yoo-Geun Ham^3^, Jin Young Kim^1^ and Daehyun Kang^1*^

*^1^Climate and Environmental Research Institute, Korea Institute of Science and Technology, Seoul, South Korea*

*^2^National Electric Power Control Center, Korea Power Exchange, Naju, South Korea*

*^3^Department of Environmental Planning, Graduate School of Environmental Studies, Seoul National University, Seoul, South Korea*

Table S1 presents a comprehensive summary of the global forecasting performance for T2M and Z500 of the KARINA model at various lead times. These results are compared against an array of state-of-the-art models, namely FourCastNet, Pangu-Weather, GraphCast, and ECMWF HRES. This specificity facilitates a more straightforward comparison with established benchmarks, thereby enhancing the clarity and relevance of the findings within the context of global weather forecasting advancements. Despite being trained on a significantly lower resolution than its counterparts, KARINA demonstrated performance that was not only comparable but in some cases superior to the other models across different lead times. This outcome suggests the reliability and efficiency of the KARINA model.

Table S1: Globally averaged latitude-weighted RMSE against ERA5 for the evaluation in 2018. RMSE for the other models was obtained from the WeatherBench2.Climatology indicates long-term averaged value for 1995-2015 with the same calendar day for the validation. The best and second-best results in each column are highlighted in bold.

| Z**500 (m^2^s^-2^)** | **Day 1** | **Day 2** | **Day 3** | **Day 4** | **Day 5** | **Day 6** | **Day 7** |
| --- | --- | --- | --- | --- | --- | --- | --- |
| GraphCast | **27.83844** | **59.21818** | **103.4287** | **163.4913** | **240.1245** | **330.5821** | **428.6702** |
| Pangu-Weather | 30.22245 | 64.63741 | 111.026 | 175.2623 | 258.0473 | 356.0197 | 460.9378 |
| IFS HRES | **32.16641** | 60.95377 | 107.4039 | 172.7858 | 257.7638 | 359.1724 | 467.5515 |
| IFS ENSmean | 32.69862 | **60.76608** | **105.9025** | **166.2882** | **240.2452** | **322.2882** | **404.097** |
| KARINA | 52.78569 | 97.44156 | 154.4761 | 223.0775 | 303.6435 | 386.2487 | 463.6588 |
| Climatology | 615.1107 | 614.8515 | 613.9937 | 615.0744 | 615.9773 | 615.3551 | 616.2557 |
| T**2M (K)** | **Day 1** | **Day 2** | **Day 3** | **Day 4** | **Day 5** | **Day 6** | **Day 7** |
| GraphCast | **0.444758** | **0.605712** | **0.758265** | **0.94009** | **1.164088** | **1.422683** | 1.697845 |
| Pangu-Weather | 0.534533 | 0.71695 | 0.88807 | 1.091347 | 1.3316 | 1.603981 | 1.887353 |
| IFS HRES | 0.808069 | 0.912112 | 1.040498 | 1.197784 | 1.400309 | 1.646615 | 1.912691 |
| IFS ENSmean | 0.856941 | 0.898555 | 1.012807 | 1.143886 | 1.300364 | 1.478012 | **1.660006** |
| KARINA | **0.482681** | **0.630802** | **0.778673** | **0.959663** | **1.156998** | **1.355944** | **1.550874** |
| Climatology | 1.831275 | 1.830474 | 1.829005 | 1.828905 | 1.827111 | 1.823755 | 1.826199 |

Table. S2 further investigated the influence of different kernel sizes in the Stem Layer. Building on existing studies that experimented with the FourCastNet model to enhance its performance, both our research and these studies have found that a smaller patch size leads to better outcomes. For instance, Cheon et al. identified a 1x1 patch size as optimal for the 2.5° resolution data^1^. Our results supported this conclusion by demonstrating that the model performed substantially better with smaller kernels, as they focused on finer spatial details, especially when dealing with lower-resolution data ^1,2^.

Table S2. Same as Table S1, except for the RMSE of KARINA experiments and varied kernel size in the stem layer. KARINA w/o both denotes KARINA model without both GeoCyclic Padding and SENet. The best and second-best results in each column are highlighted in bold.

| Z**500 (m2s-2)** | **Day 1** | **Day 2** | **Day 3** | **Day 4** | **Day 5** | **Day 6** | **Day 7** |
| --- | --- | --- | --- | --- | --- | --- | --- |
| KARINA | **52.7856** | **97.44156** | **154.4761** | **223.0775** | **303.6435** | **386.2487** | **463.6588** |
| KARINA w/o Pad | 65.9577 | 134.7612 | 219.5569 | 307.5233 | 399.6926 | 482.6942 | 553.928 |
| KARINA w/o SENet | 60.8064 | 112.1798 | 174.9202 | 248.5538 | 332.2471 | 414.4274 | 491.5129 |
| KARINA w/o both | 78.537 | 159.1034 | 252.1735 | 346.5416 | 438.6842 | 515.5434 | 581.8939 |
| KARINA kernel 5x5 | 57.3606 | 103.0708 | 161.6738 | 232.4983 | 314.701 | 396.7027 | 471.7742 |
| KARINA kernel 7x7 | **55.4845** | **100.2935** | **157.2569** | **224.9177** | **305.7518** | **388.6726** | **463.9491** |
| T**2M (K)** | **Day 1** | **Day 2** | **Day 3** | **Day 4** | **Day 5** | **Day 6** | **Day 7** |
| KARINA | 0.48268 | **0.630802** | **0.778673** | **0.959663** | **1.156998** | **1.355944** | **1.550874** |
| KARINA w/o Pad | 0.503863 | 0.711681 | 0.944736 | 1.210786 | 1.474297 | 1.719928 | 1.937172 |
| KARINA w/o SENet | **0.480118** | **0.63536** | **0.79646** | **0.987325** | **1.192336** | **1.399853** | **1.592042** |
| KARINA w/o both | 0.513939 | 0.706354 | 0.928033 | 1.176686 | 1.417018 | 1.631659 | 1.816150 |
| KARINA kernel 5x5 | **0.482499** | 0.652018 | 0.825798 | 1.031518 | 1.25275 | 1.473521 | 1.68243 |
| KARINA kernel 7x7 | 0.497753 | 0.659697 | 0.826425 | 1.016939 | 1.22497 | 1.444738 | 1.640302 |

Table S3. List of Atmospheric Variables ERA5 used in KARINA, Corresponding Short Names, Vertical Pressure Levels in Hectopascals (hPa), and Standard Units of Measurement

| **Variable name** | **Short name** | **Vertical levels (hPa)** | **Units** |
| --- | --- | --- | --- |
| Zonal wind | U | 1000,925,850,800,700,600,500,400,300,200,100,50 | m/s |
| Meridional wind | V | 1000,925,850,800,700,600,500,400,300,200,100,50 | m/s |
| Temperature | T | 1000,925,850,800,700,600,500,400,300,200,100,50 | K |
| Specific humidity | Q | 1000,925,850,800,700,600,500,400,300,200,100,50 | kg/kg |
| Geopotential | Z | 1000,925,850,800,700,600,500,400,300,200,100,50 | $m^{2}/s^{2}$ |
| 2m temperature | T2M |  | K |
| Mean sea level pressure | MSLP |  | Pa |
| Surface air pressure | SP |  | Pa |
| Total column vertically-integrated water vapor | TCWV |  | Kg/$m^{2}$ |
| Skin temperature | SKT |  | K |
| TOA incident solar radiation | TISR |  | J/$m^{2}$ |

Table S4: Overview of Essential Hyperparameters in the KARINA Model for Global Weather Forecasting (Note: "dt" represents the prediction lead time, quantified as the number of steps ahead).

| Hyperparameter | Value |
| --- | --- |
| loss | l2 |
| Learning rate (LR) | 0.001 |
| scheduler | CosineAnnealingLR |
| dt | 1 |
| Number of in_channels | 67 |
| Number of out_channels | 67 |
| normalization | Z-score |
| Optimizer type | AdamW |
| max_epochs | 150 |
| Batch size | 8 |
| Number of Parameters | 35M |
| Per-GPU Memory | 33GB |

Table S5. Comparative Analysis of Training Times and Hardware Specifications for Deep Learning Models. All models listed, with the exception of KARINA (trained at 2.5° resolution), were trained on data with a 0.25° spatial resolution.

| **Model** | **Number of GPUs** | **Training Time** |
| --- | --- | --- |
| FuXi^3^ | 8 Nvidia A100 GPUs. | 30 hrs |
| Fengwu^4^ | 32 Nvidia A100 GPUs | 17 days |
| FCN^5^ | 64 Nvidia A100 GPUs. | 16 hrs |
| GraphCast^6^ | 32 single Google Cloud TPU v4 | 4 weeks |
| Pangu Weather^7^ | 192 NVIDIA Tesla-V100 GPUs. | 64 days |
| KARINA^8^ | 4 Nvidia A100 GPUs | 12 hrs |

###


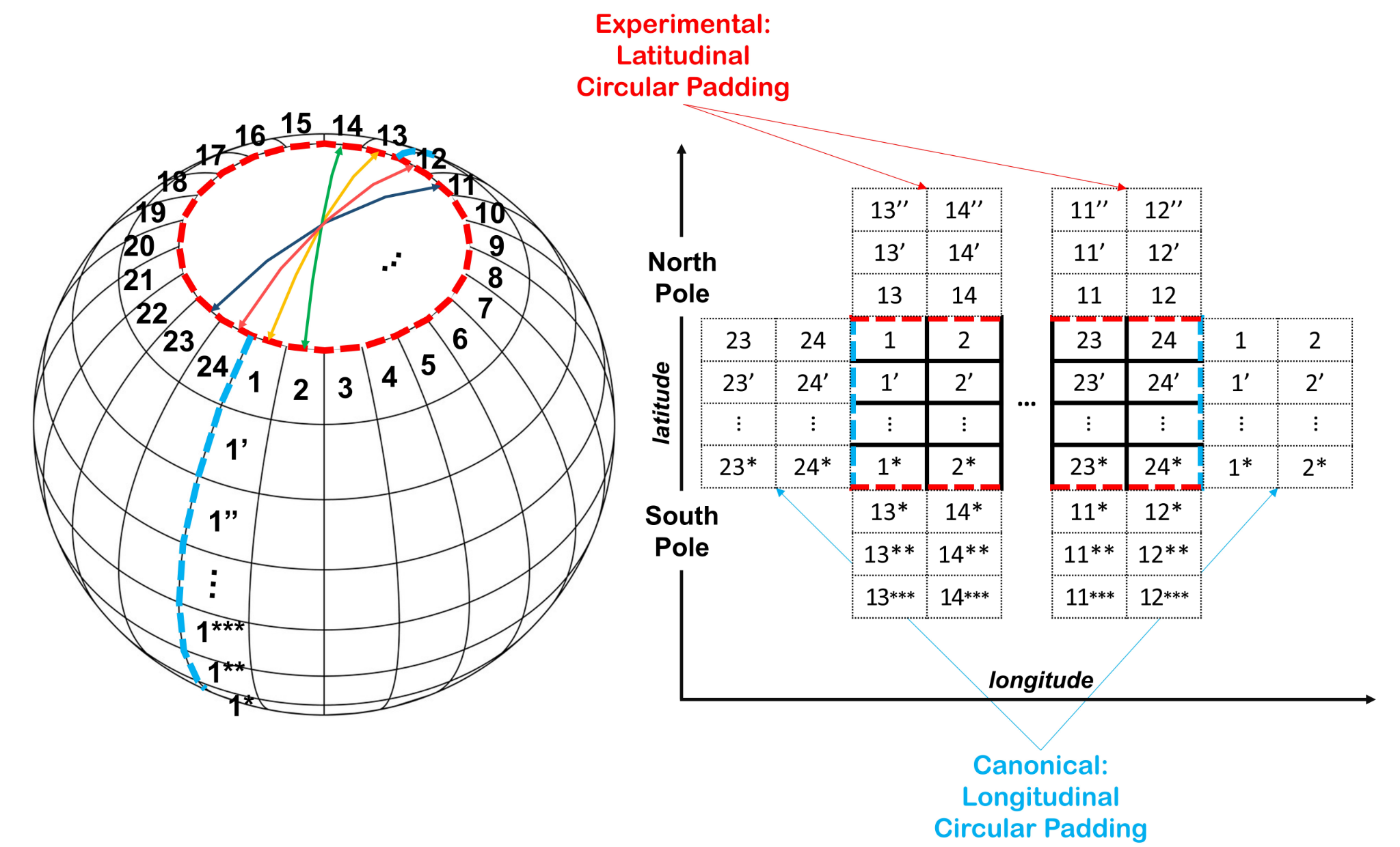


Figure S1. Description of GeoCyclic Padding minimizes projection distortions by circularly padding the edges and reordering at the poles to ensure longitudinal continuity in geographic data such as the ERA5 dataset.


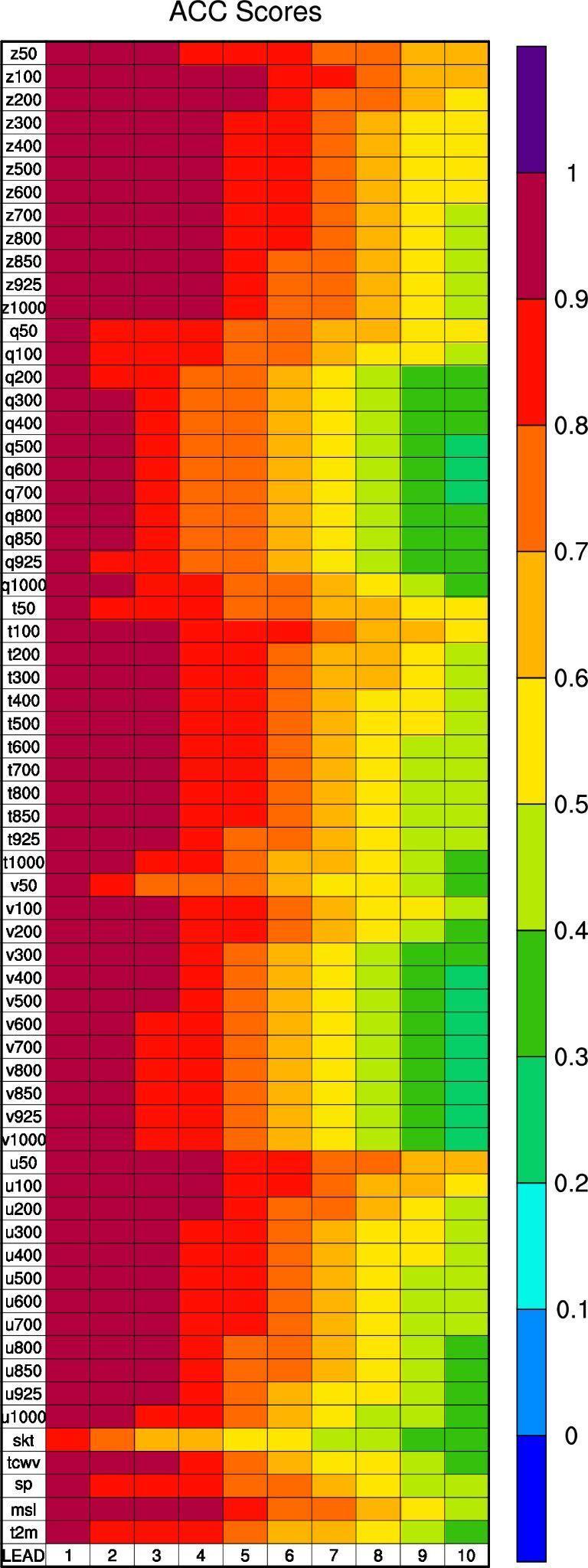


Figure S2: ACC between KARINA and ERA5 of all variables until forecast day 10

**Figure S2: A coherent skillful prediction of all variables**

Figure S2 illustrates the ACC skill scores for the various predictive variables within our model, extending through a 10-day forecast period. The proposed model demonstrated competent forecasting capabilities across all variables within the first week. Specifically, it exhibited enhanced proficiency for pressure-level variables, with particularly adept forecasting observed in the upper troposphere compared to surface levels. This result indicated that additional effort is necessary to improve the surface boundary condition in the upcoming models.


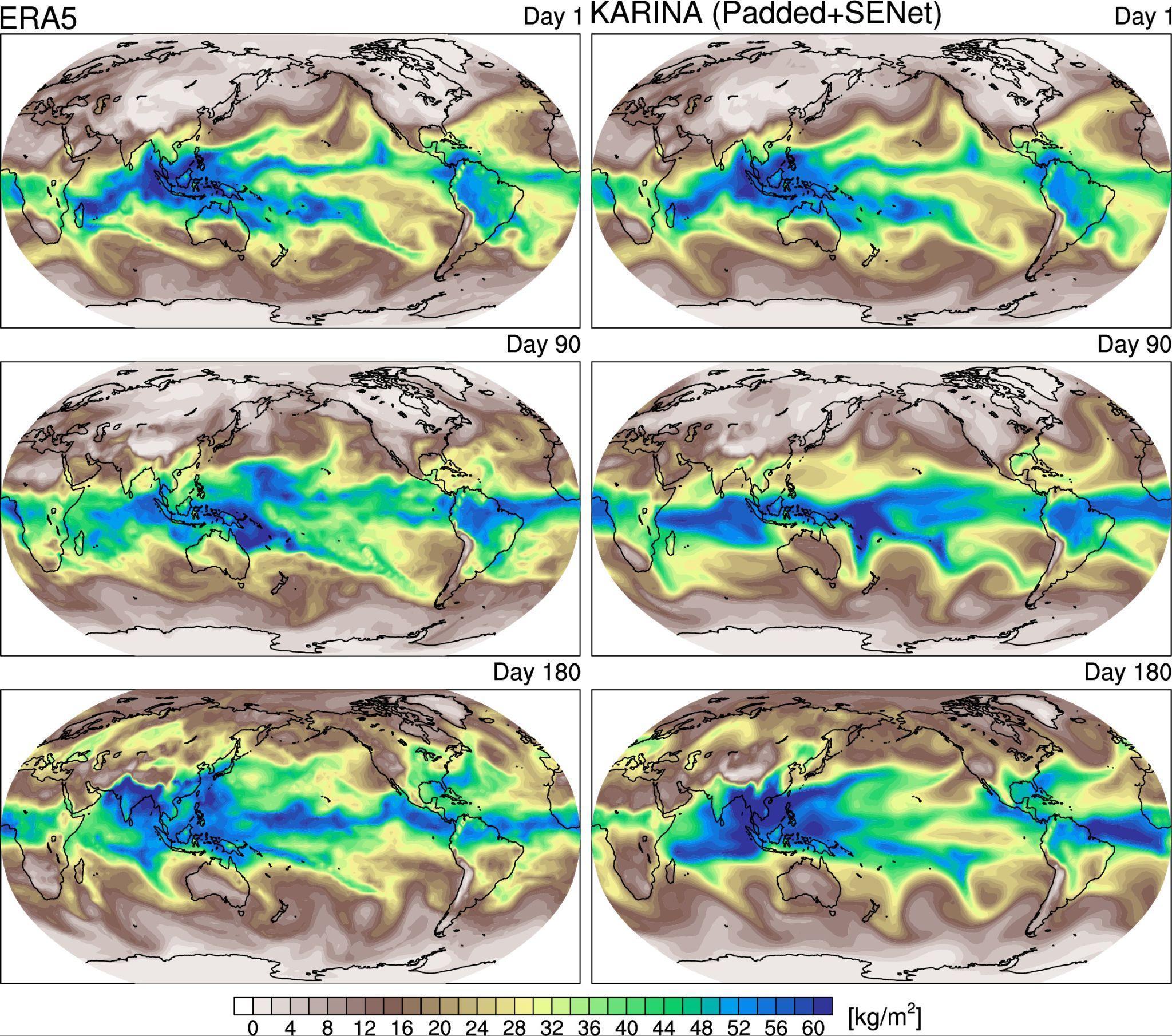


Figure S3. Horizontal distribution of total column vertically-integrated water vapor (TCWV) variable obtained from the different autoregressive inference steps (day 1, day 90, and day 180) of KARINA beginning on Jan. 1st, 2018, and ERA5 on the same calendar day. The maps were generated using the NCAR Command Language (version 6.6.2; http://dx.doi.org/10.5065/D6WD3XH5)

**Figure S3: Stable autoregressive inference**

Occasionally, a repetitive autoregressive inference of global weather prediction is unstable in some models, which could diminish the efficacy of data-driven models for reliable long-range forecasts. Some models have shown stable inference even until a year^9,10^, while many models have not demonstrated their stability. Fig. S3 shows the TCWV variable, total water vapor amount in the atmospheric column, obtained from the autoregressive inference of KARINA beginning on Jan. 1st, 2018. Compared with ERA5 as ground truth, long-range prediction of KARINA revealed a realistic distribution of global atmospheric water vapor. Although detailed patterns on days 90 and 180 differed from ERA5 because of the less predictability in these forecast days, KARINA reproduced a stable global pattern over six months.


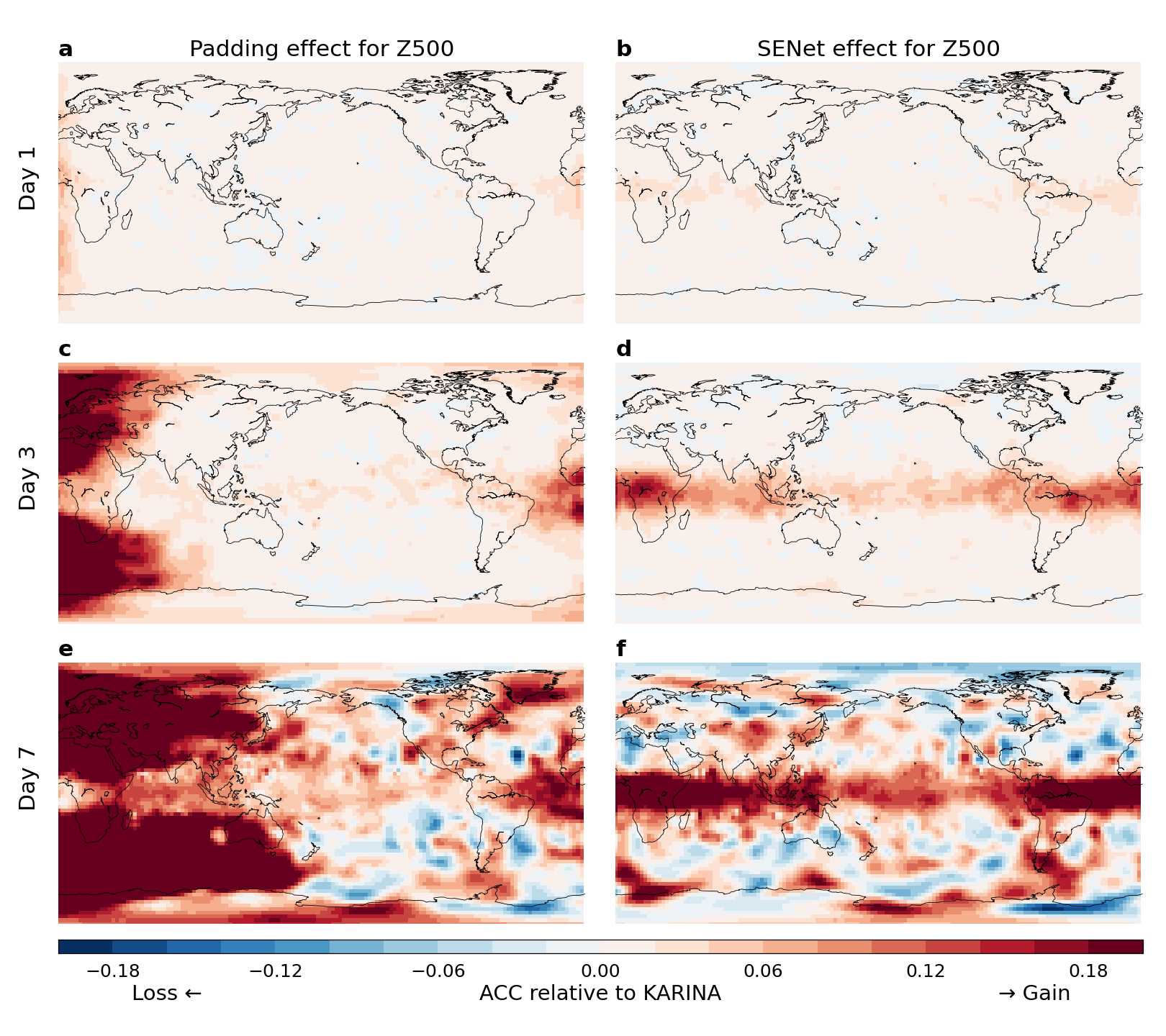


Figure S4. Effect of GeoCyclic Padding of SENet with increasing forecast days. The region-dependent effects of GeoCyclic Padding and SENet on 1-, 3-, and 5-day forecasting performance of KARINA for Z500. a, c, and e (first column), ACC relative to KARINA of KARINA w/o pad (i.e., KARINA without GeoCyclic Padding) for Z500 on 1-, 3-, and 5-day forecast, respectively. b, d, and f (second column), Same as a, c, and e (first column), but for KARINA w/o SENet. The maps were generated using the Python Matplotlib (version 3.7.2; http://matplotlib.org/)

**Figure S4: Effect of GeoCyclic Padding and SENet**

The Figure S4 displays gradual propagation of error improvement by GeoCyclic Padding and SENet.


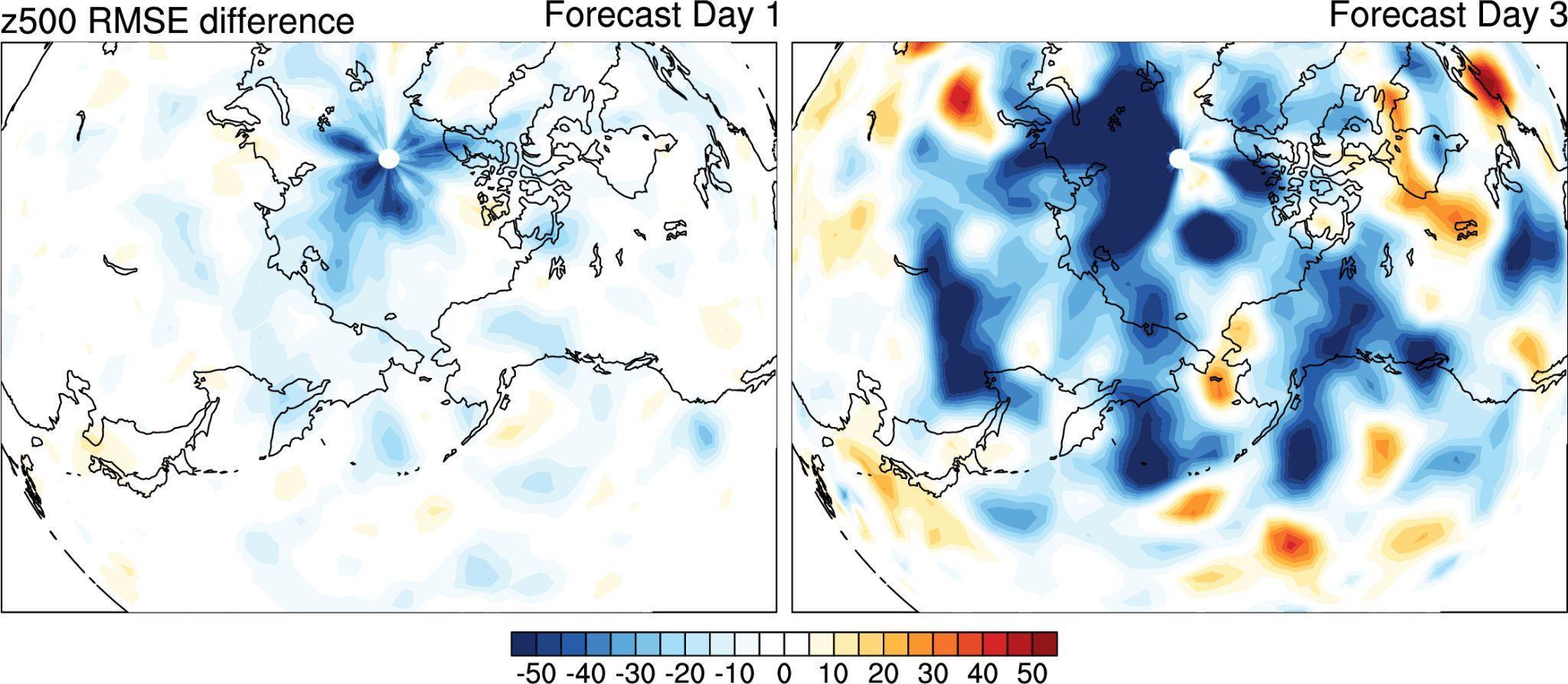


Figure S5. Horizontal distribution of RMSE improvement contributed by the latitudinal edge of GeoCcclic Padding, which is defined as the RMSE difference of Z500 between GeoCyclic Padding and Circular Padding during the test period for (left) 1- and (right) 3-day forecast. The maps were generated using the NCAR Command Language (version 6.6.2; http://dx.doi.org/10.5065/D6WD3XH5)

**Figure S5: GeoCyclic Padding Effect at the Poles**

GeoCyclic Padding proposed in this study covers both the latitudinal and longitudinal edges of the image, which showed a marked improvement in global weather prediction. Here, we separated the effect of GeoCyclic padding at the poles to figure out how it performs effectively in the polar regions. We employed 'Circular Padding,' which mirrors the concept of GeoCyclic Padding along the longitudinal axis. This method is distinguished by using zero padding at the northern and southern edges. The partial effect of GeoCyclic Padding at the latitudinal edge was evaluated by a difference of RMSE between GeoCyclic Padding and Circular Padding in Fig. S5. The partial GeoCyclic Padding effect revealed a noticeable error reduction near the pole on forecast day 1. On day 3, this improvement spread to mid-latitude during autoregressive inference with amplified accuracy. This result demonstrated the effective contribution of latitudinal edges in GeoCyclic Padding, distinguished from Circular Padding.


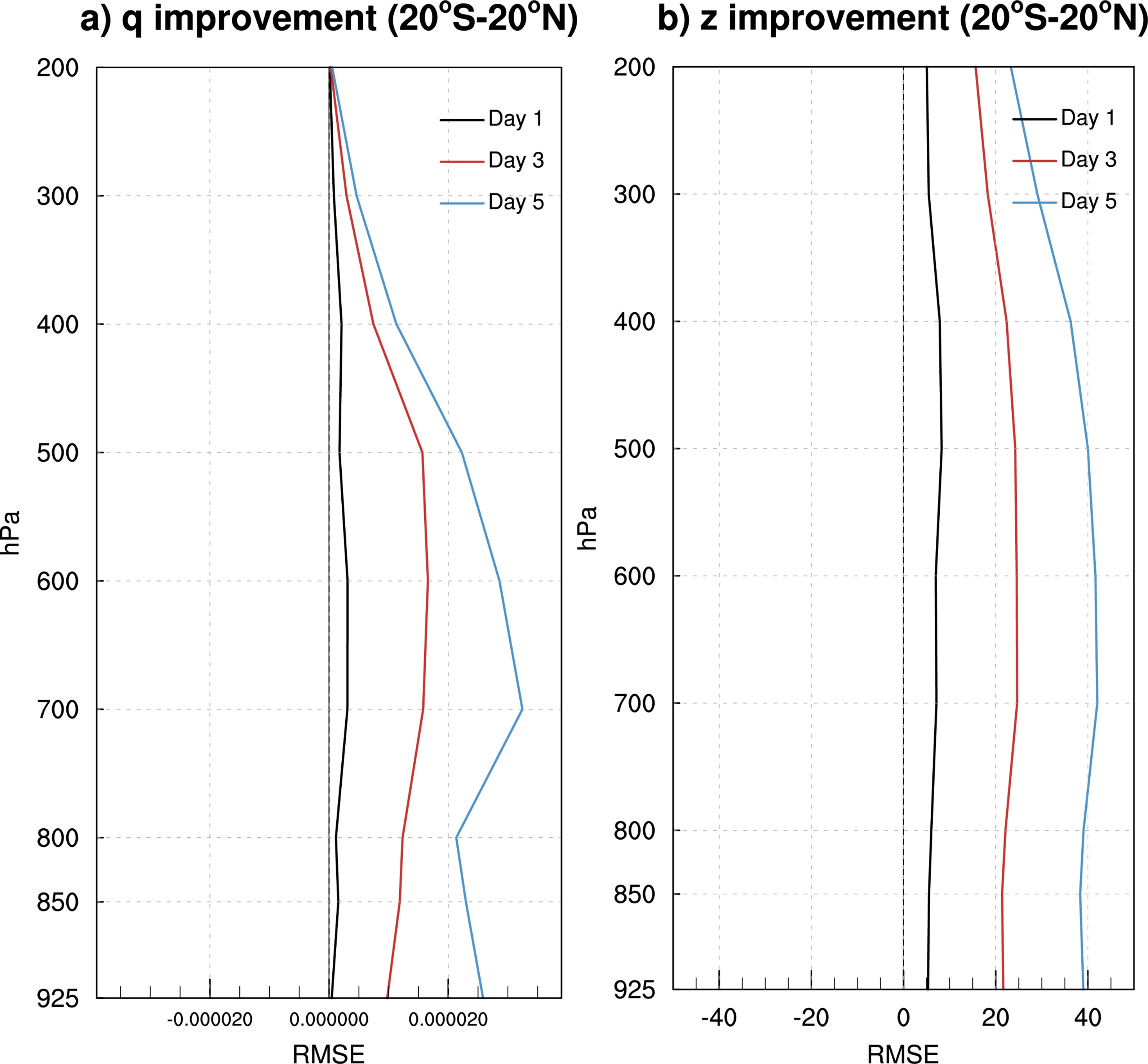


Figure S6. Difference of area-averaged RMSE (20S-20N) between KARINA and KARINA w/o SENet. a) Vertical profile of specific humidity (Q) and b) geopotential (Z) at forecast day 1 (black), day 3 (red), and day 5 (blue).

**Figure S6: The RMSE improvement from SENet effect in the tropics**

The skill improvement in the tropics suggests that SENet channel integration enhances the model's ability to represent atmospheric column processes more realistically. Specifically, the improvements for both specific humidity and geopotential are most prominent between 700 and 500 hPa (Fig. S6), an atmospheric layer where moist convection is a significant driver of perturbations and diabatic heating.

**
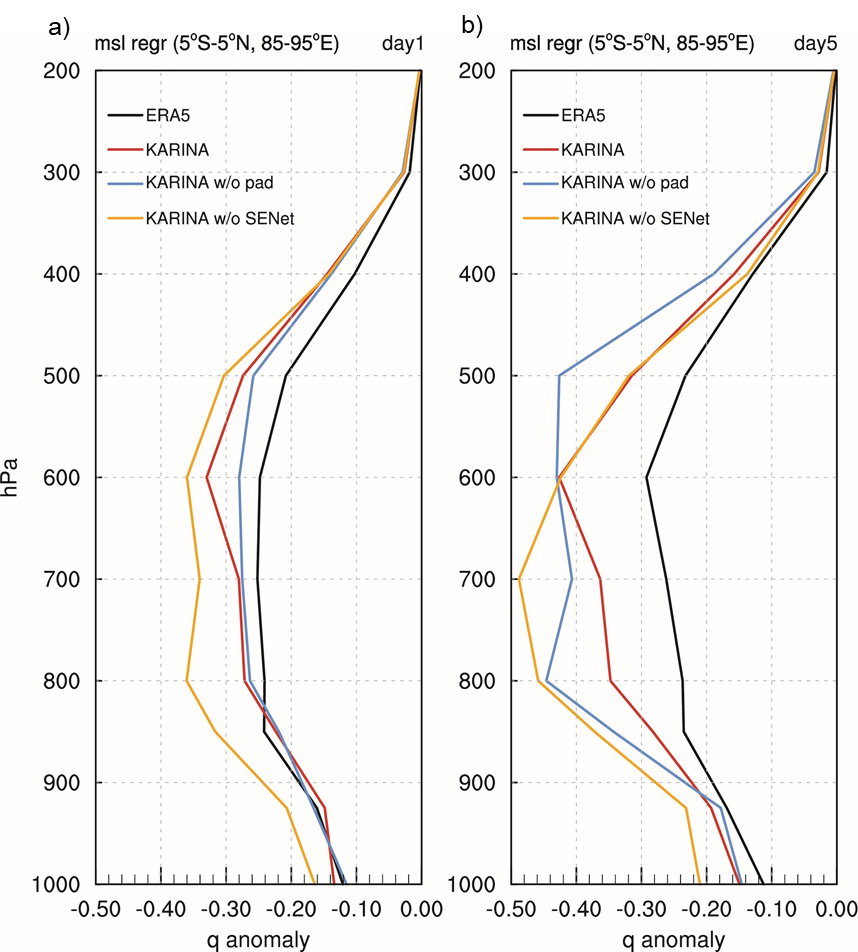
**

Figure S7. Regressed vertical profile of specific humidity (Q) onto mean sea-level pressure (MSLP) in the tropical Indian Ocean (85-95E; 5S-5N) for the forecast a,) day 1 and b,) day 5. The regression is performed with daily anomalies of all initial conditions in 2018 and the corresponding days for ERA5. Each line refers to ERA5 (black), KARINA (red), KARINA w/o pad (blue), and KARINA w/o SENet (orange).

**Figure S7: SENet effect in the equatorial atmospheric column**

To examine the relationship among atmospheric columns in the tropical convective region, Fig. S7 compares the vertical profile of specific humidity linearly regressed onto mean sea level pressure (MSLP) in the tropical Indian Ocean (85-95E; 5S-5N). The negative relationship between specific humidity in the mid-to low-troposphere (850-500 hPa) and MSLP corresponds to a larger moisture amount in a convective region (i.e., lower pressure). On forecast day 1, both KARINA and KARINA w/o pad reproduce the vertical profile realistically, but KARINA w/o SENet overestimates the moisture-pressure relationship. On forecast day 5, the relationships among the KARINA models are consistent, while KARINA w/o pad also exhibits an overestimation of the relationship, which can be explained by a spreading padding effect from edges to the Indian Ocean with increasing forecast days. This result indicates that the inter-channel relationship in the atmospheric column is remarkably improved by employing the SENet.


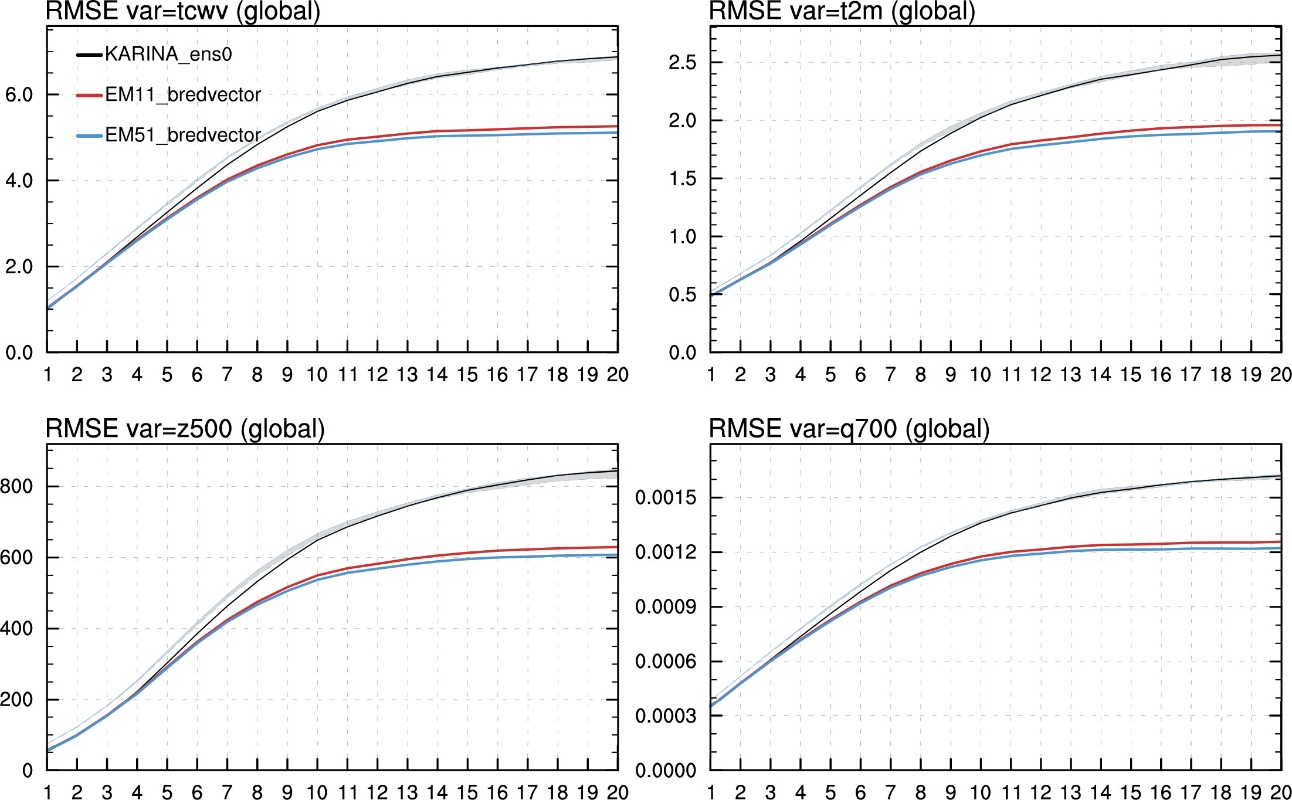


Figure S8. The globally averaged latitude-weighted forecast skills in 2018. Colors indicate KARINA baseline single member (black), 11-member ensemble mean (red), 51-member ensemble mean (blue), and the other individual members (gray shaded). Panels denote each variable: TCWV, T2M, Z500, and Q700.

**Figure S8: Ensemble prediction with bred vector perturbation**

Fig. S8 shows that multi-ensemble prediction effectively reduces forecast of KARINA as in dynamical prediction systems. The perturbed initial conditions for ensemble prediction were generated by a bred vector method as in Mahesh et al.^11^. The Z500 of each initial state was randomly perturbed, then the breeding was performed by KARINA for the random initial condition. The resulting initial condition indicate the dynamically reliable perturbation field for the entire variables.


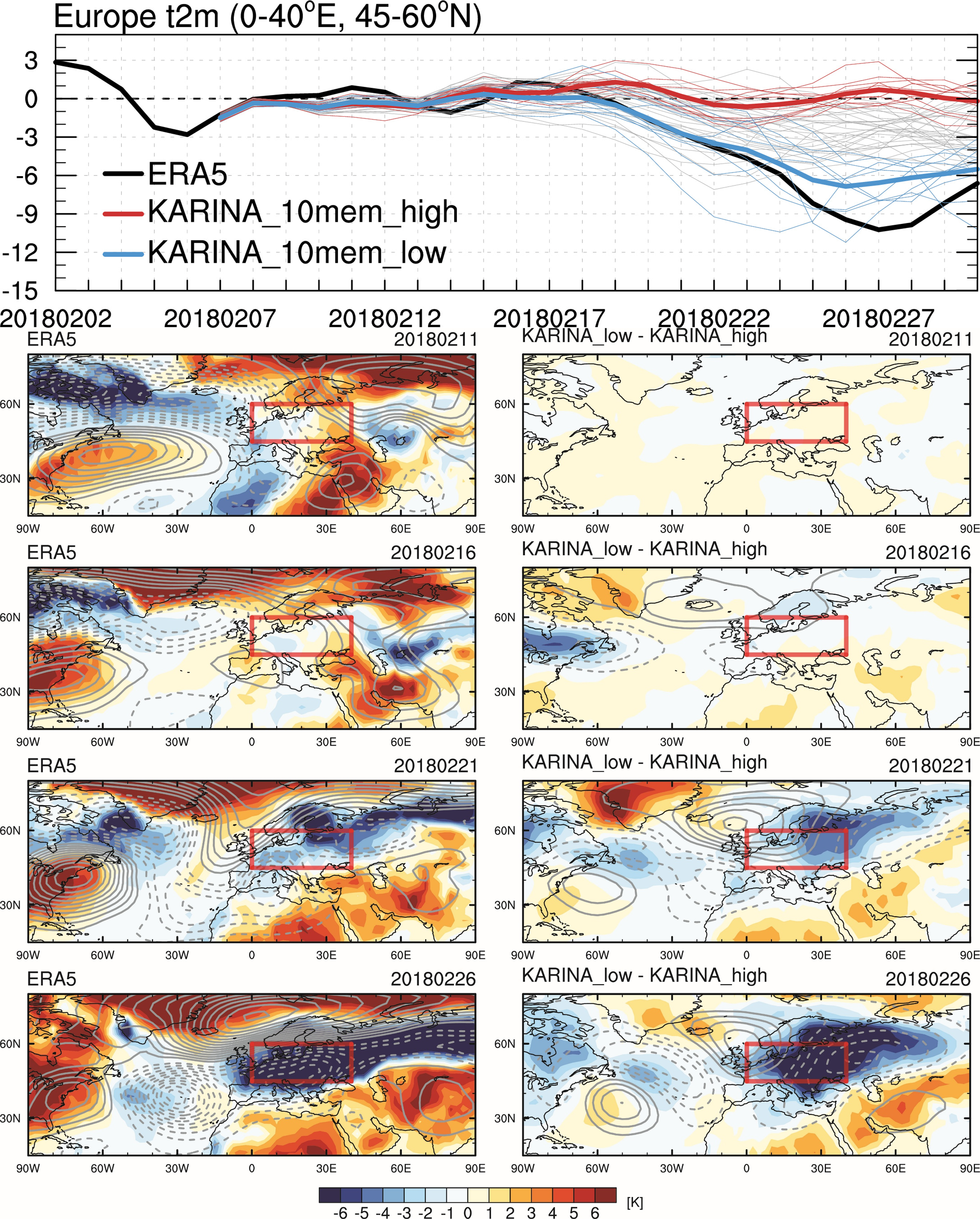


Figure S9. (top) T2M averaged in Europe (0-40E, 45-60N) for ERA5 (black) and KARINA initialized on 20180206 for 51 ensemble members (thin lines), and each 10-member group of the highest and lowest T2M prediction on 0226-0228 (KARINA_high and KARINA_low, respectively). (Bottom left) Evolution of Z500 (contour) and T2M (shaded) for ERA5, and (Bottom Right) Difference between KARINA_low and KARINA_high). The maps were generated using the NCAR Command Language (version 6.6.2; http://dx.doi.org/10.5065/D6WD3XH5)

**Figure S9: Process diagnostics for cold waves from ensemble spread**

Fig. S9 shows that multi-ensemble spread for the European extreme cold events in 2018. The difference in ensemble groups suggest that the predicted cold wave was developed from the wave train passing the North Atlantic Ocean

### Reference

1. Cheon, M., Kang, D., Choi, Y.-H. & Kang, S.-Y. Advancing data-driven weather Forecasting: Time-Sliding data augmentation of ERA5. Preprint at https://arxiv.org/abs/2402.08185 (2024).
2. Guo, E. et al. FourCastNEXT:. Optimizing FourCastNet training for limited compute. Preprint at https://arxiv.org/abs/2401.05584 (2024).
3. Chen, L. et al. FuXi: a cascade machine learning forecasting system for 15-day global weather forecast. Npj Climate and Atmospheric Science 6, (2023).
4. Chen, K. et al. FengWu: Pushing the Skillful Global Medium-range Weather Forecast beyond 10 Days Lead. Preprint at https://arxiv.org/abs/2304.02948 (2023).
5. Pathak, J. et al. FourCastNet: A Global Data-driven High-resolution Weather Model using Adaptive Fourier Neural Operators. arXiv.org https://arxiv.org/abs/2202.11214 (2022).
6. Lam, R. et al. Learning skillful medium-range global weather forecasting. Science 382, 1416–1421 (2023). Bi, K. et al.
7. Accurate medium-range global weather forecasting with 3D neural networks. Nature 619, 533–538 (2023).
8. Cheon, M. et al. KARINA: an efficient deep learning model for global weather forecast. Preprint at https://arxiv.org/abs/2403.10555 (2024).
9. Weyn, J. A., Durran, D. R., Caruana, R. & Cresswell‐Clay, N. Sub‐Seasonal forecasting with a large ensemble of Deep‐Learning weather prediction models. Journal of Advances in Modeling Earth Systems 13, (2021).
10. Bonev, B. et al. Spherical fourier Neural Operators: learning stable dynamics on the sphere. Preprint at https://arxiv.org/abs/2306.03838 (2023).
11. Mahesh, A., et al. Huge ensembles part i: Design of ensemble weather forecasts using spherical fourier neural operators. Preprint at https://arxiv.org/abs/2408.03100 (2024).
